# Development and promotion of a national website to improve dissemination of information related to the prevention of mother-to-child HIV transmission (PMTCT) in Tanzania

**DOI:** 10.1186/s12889-015-2422-x

**Published:** 2015-10-22

**Authors:** Gudila Stephan, Mary Jo Hoyt, Deborah S. Storm, Sylvia Shirima, Charles Matiko, Emmanuel Matechi

**Affiliations:** FXBT Health, Dar-es-Salaam, Tanzania; François-Xavier Bagnoud Center, School of Nursing, Rutgers, The State University of New Jersey, Newark, New Jersey USA

## Abstract

**Background:**

Websites that address national public health issues provide an important mechanism to improve health education and services in resource limited countries. This article describes the development, promotion and initial evaluation of a national website to increase access to information and resources about prevention of mother-to-child transmission of HIV (PMTCT) among healthcare workers and PMTCT stakeholders in Tanzania.

**Methods:**

A participatory approach, involving the Tanzania Ministry of Health and Social Welfare (MOHSW) and key PMTCT stakeholders, was used to develop and manage the online PMTCT National Resource Center (NRC), http://pmtct.or.tz/. The website was created with a content management system software system that does not require advanced computer skills and facilitates content updates and site management. The PMTCT NRC hosts related regularly updated PMTCT-related news, resources and publications. Website implementation, access and performance were evaluated over two years using Google Analytics data about visits, page views, downloads, bounce rates and location of visitors, supplemented by anecdotal feedback.

**Results:**

Following its launch in July 2013, the PMTCT NRC website received a total of 28,400 visits, with 66,463 page views, over 2 years; 30 % of visits were from returning visitors. During year 1, visits increased by 80 % from the first to second 6 month period and then declined slightly (9–11 %) but remained stable in Year 2. Monthly visits spiked by about 70 % during October 2013 and January 2014 in response to the release and promotion of revised national PMTCT guidelines and training manuals. The majority of visitors came from primarily urban areas in Tanzania (50 %) and from other African countries (16 %). By year 2, over one-third of visitors used mobile devices to access the site.

**Conclusions:**

The successfully implemented PMTCT NRC website provides centralized, easily accessed information designed to address the needs of clinicians, educators and program partners in Tanzania. Ongoing involvement of the MOHSW and key stakeholders are essential ensure the website’s growth, effectiveness and sustainability. Additional efforts are needed to expand use of the PMTCT NRC throughout the country. Future evaluations should examine the role of the website in supporting implementation of national PMTCT guidelines and services in Tanzania.

## Background

The Internet and electronic information and communication technologies have been shown to improve dissemination of public health information and have facilitated collaboration and cooperation among healthcare workers, particularly in developing countries [1]. Growth in internet access globally has enabled increasing numbers of healthcare workers to access online health information, including practice guidelines, and to communicate with each other [2–5]. Health communication websites have been shown to be feasible and successful in promoting access to health information [6, 7]. Identified benefits of social media include increased interactions and information sharing, peer support, greater access to health information and potential to influence health policy [8, 9].

In Tanzania, as in other developing countries, information and communication technologies have not been systematically used to improve the population’s health [10]. The Ministry of Health and Social Welfare (MOHSW) has taken deliberate actions to address this by defining a national e-health vision, mission and goals and the pathway to reach them in the Tanzania National e-Health Strategy 2013 – 2018 [11]. One of the strategic objectives of this plan is to support improved multi-way communication and sharing of information among clinicians, patients and caregivers within the health sector and across partner agencies. According to the World Bank, Tanzania has seen a significant growth in internet use among the general population from 0.1 % in 2000 to 13.1 % in 2012 [12, 13]. Increasing availability of internet access and evidence about the importance of electronic health resources support the need to establish and maintain online health information repositories for healthcare workers and patients. This article describes the development, promotion and initial evaluation of the prevention of mother-to-child transmission of HIV (PMTCT) National Resource Center (NRC) website (http://pmtct.or.tz/) which was designed to increase access to information and resources and to serve as a communication platform for the continuing advancement of PMTCT services in Tanzania.

### Rationale for the development of the Tanzania PMTCT NRC

Prevention of perinatal HIV transmission is a global commitment actively shared by the MOHSW. In Tanzania, HIV prevalence among women who attend antenatal care was 6.9 % in 2008 [14]. Although PMTCT services are widely available, approximately 15 % of infants born to HIV positive mothers become HIV infected [15]. In an effort to address these problems, the MOHSW has adopted the Joint United Nations Programme on HIV and AIDS (UNAIDS) Global Plan Towards the Elimination of New HIV Infections Among Children by 2015 and Keeping Their Mothers Alive [16] and follows World Health Organization (WHO) recommendations for PMTCT [17]. In 2012, WHO updated its PMTCT guidelines to include Option B + —a single, universal antiretroviral regimen for PMTCT and HIV treatment—and in 2013 established Option B+ as the recommended option in the WHO *Consolidated guidelines on the use of antiretroviral drugs for treating and preventing HIV infection* [18]. With Option B+, all HIV-positive pregnant and breastfeeding women are provided with lifelong combination antiretroviral treatment (cART), regardless of the CD4 count, clinical disease stage, or gestational age of the fetus. Tanzania is currently implementing Option B+ in order to accelerate progress towards the goal of eliminating new HIV infections in children and keeping their mothers alive and healthy.

The MOHSW works with a group of more than ten partner organizations to build health system capacity and deliver PMTCT services in all regions. National PMTCT guidelines and associated training manuals provide core resources for PMTCT services and healthcare worker training. Typically, when guidelines and related resources are developed, hardcopies are printed and disseminated to health facilities by the MOHSW. However, anecdotal experience and published studies have reported shortages of these types of materials in health facilities [19, 20]. One strategy to address this problem is to create electronic versions that are readily available through the Internet. In anticipation of the release of the 2013 Tanzania National Guidelines for Comprehensive Care Services for PMTCT and Keeping Mothers Alive (Option B+) and the National Training Refresher Package PMTCT Manuals (Option B+), a decision was made to create a user friendly, PMTCT-focused website that would support the dissemination of PMTCT resources and information and complement existing or planned websites. The MOHSW website (http://www.moh.go.tz) addresses a broad range of information and resources about national health and social welfare policies. The National AIDS Control Programme (NACP) website (http://www.nacp.go.tz) was launched in 2006 to share materials related to HIV and sexually transmitted infections (STIs). The Reproductive and Child Health Section (RCHS) website (http://www.rchs.go.tz/index.php/en), which was launched after the PMTCT NRC, focuses on comprehensive reproductive and child health services. However, these websites address multiple topics and were not designed to provide comprehensive information and resources on PMTCT. In addition, they have limited search capacity, requiring users to browse the site to locate PMTCT specific materials. It can also be challenging for large, governmental websites to keep pace with frequent changes in a single topic area, such as PMTCT. These issues contributed to the recognized need for a centralized, online information repository for PMTCT-related news, resources, best practices, reports and publications.

## Methods

### Development and evaluation of the PMTCT National Resource Center (NRC) website

The François-Xavier Bagnoud Center Tanzania (now FXBT Health), a technical assistance partner of the MOHSW, utilized a participatory approach to create the online PMTCT NRC website (http://pmtct.or.tz) in collaboration with the MOHSW and PMTCT stakeholders. The MOHSW and key stakeholders were involved in all stages of development to ensure ownership and sustainability of the website. A website advisory board with representatives from PMTCT supporting organizations, MOHSW and the U.S. Centers for Disease Control and Prevention (CDC) was established and meets quarterly to discuss and provide advice on the development and management of the website. A specific plan of activities, monitoring indicators, outputs and outcomes, summarized in Table 1, was created to support effective development, implementation and evaluation of the website.

**Table 1:** Tanzania PMTCT webpage monitoring data. Overview of the Tanzania Prevention of Mother-to-Child Transmission of HIV (PMTCT) National Resource Center (NRC) website development and implementation: activities, monitoring indicators, outputs, and outcomes

Website development and implementation activities	Pre-launch monitoring indicators and outputs	Post-launch monitoring indicators and outputs	Targeted outputs and outcomes
• Engage stakeholders, form Advisory Committee • Identify appropriate staff and technical consultants, e.g., website/social media designer, host, manager • Analyze the target audience and their requirements • Develop site architecture and create website, e.g., content development and placement, wireframes and mock-ups • Conduct usability testing; review and revise • Migrate content and implement CMS^a^ • Conduct staff training on use and management of website software • Develop and implement marketing (including social media) and evaluation plans • Implement ongoing website management and updates/revisions • Conduct ongoing evaluation using Google Analytics and other strategies	• Stakeholder Advisory committee meetings and input per minutes • Cycles of usability tests and revisions completed • Web links to and from PMCT.or.tz established • Finalized site content includes key PMTCT resources, links, PMTCT partner contacts and resources (Partners catalogue), static survey, etc. • Google Analytics in place • Staff independently updating website and establishing social media presence • Website marketing materials finalized and distributed • Social media marketing campaign for launch • PMTCT website launch meeting and launch on planned date	• Google analytics data of site visitors and use, e.g., # and source of visitors, # and type of downloaded resources, # referred visitors from linked websites • Interactive discussion forum added to the website • Discussion forum activity and content • Additional PMTCT partner resources linked or posted on the website • Static survey reports • Number of Facebook likes and Twitter followers • Social media and marketing campaigns for updated PMTCT guidelines and other key resources • Ongoing site management with content updates, repair of broken web links, etc. • Proportion accessing via mobile device, utilizing Swahili version	• Growth in website use and downloads over time with spikes related to marketing campaigns and addition of key resources • Easily accessed key resources from web links, google searches, listserv, etc. • Growth in content with increased access to PMTCT partners information/resources • Increased awareness of PMTCT partners activities • Growth in listserv and social media • Strengthened communication among PMTCT Partners and with the MOHSW • Increased PMTCT knowledge among health care workers, program managers and policy makers

^a^Content management systems (CMS)

A content management system software (CMS) was used to create the website. This easy-to-use application allows anyone with basic computer knowledge and skills to manage and update the website. The website is mobile-device friendly, enabling access to resources by those who have mobile devices with internet services. A local language (Swahili) version of the website was also created to broaden the spectrum of the users. Google Analytics software was installed to monitor and observe the behavior of website visitors. Website implementation, access and performance were evaluated from July 2013 through June 2015 using Google Analytics data about key indicators such as visits, page views, downloads, bounce rates and location of visitors, supplemented by anecdotal feedback.

### Website content

**Fig. 1:**
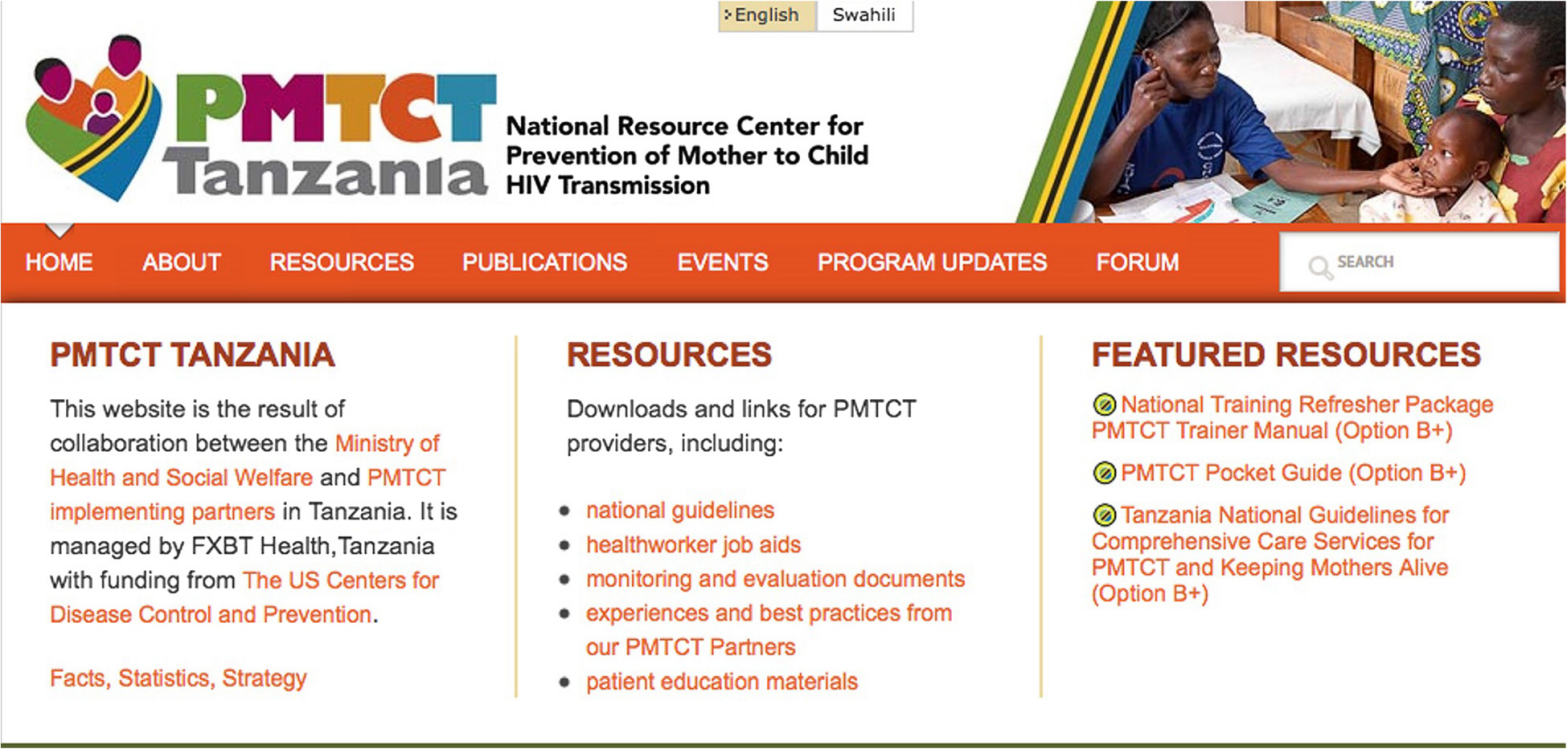
Tanzania PMTCT website homepage. The Tanzania Prevention of Mother-to-Child Transmission of HIV National Resource Center website home page (http://pmtct.or.tz/) header, illustrating some of the resources available on the website. The image on this figure was purchased with the following Rights Granted defined on the paid invoice: RIGHTS GRANTED: For EDITORIAL (NON-COMMERCIALS) USE ONLY. 100,000 maximum prints or impressions (including collateral and multimedia uses). Unlimited use on one Website permitted. Worldwide distribution permitted

The PMTCT NRC website is populated with PMTCT news, resources and publications and is updated at least twice a week. Content is categorized by type of resource and topic for ease of access. Core resources, featured resources and featured publications are promoted on the home page (Fig. 1). Sources of posted materials include MOHSW, Tanzania PMTCT partner organizations, WHO, Joint United Nations Programme on HIV and AIDS (UNAIDS) and other free online HIV/AIDS repositories such as PubMed, Medline and AIDSMAP. In order to ensure the quality of information, posted materials and information are limited to published, peer-reviewed, MOHSW-approved or known and trusted sources. The website also features a Discussion Forum where PMTCT stakeholders can exchange ideas about ongoing issues.

### Promotion of the website

Marketing materials, presentations and social media campaigns were developed to publicize and promote the PMTCT website. Post cards, posters, bags and pens with the website logo and uniform resource locator (URL) were distributed to the MOHSW, PMTCT partners and other stakeholders so they could share them with healthcare workers and other constituents. Promotional materials were also distributed to Health Sciences Institutes—public and private learning institutions for medicine, nursing and other allied health disciplines. An official launch event was held for the debut of the PMTCT website in July 2013. The website has been presented during PMTCT stakeholders’ meetings and other meetings held by the MOHSW and at the 3^rd^ Tanzania National Quality Improvement Forum in November 2013. A booth was secured at the Tanzania National Family Planning Conference to display the website and distribute website marketing materials. Cross-linkages were created to promote the online PMTCT NRC through websites of the MOHSW and its institutions, the Health Sciences Institutes and PMTCT supporting organizations.

A social media strategy was developed and implemented as a mechanism for dissemination of PMTCT information and as a marketing tool for the website. The strategy includes an email listserv, a Facebook page, Twitter account linked to the website and the online Discussion Forum. A promotional campaign using social media was conducted to increase awareness of and access to Option B+ materials following the release of updated national PMTCT guidelines and PMTCT refresher training package in October 2013 and January 2014, respectively. The special links created for these resources were shared through Facebook health professionals’ pages and groups, health professional emailing groups and emails to regional and district HIV/AIDS program managers.

## Results

**Fig. 2:**
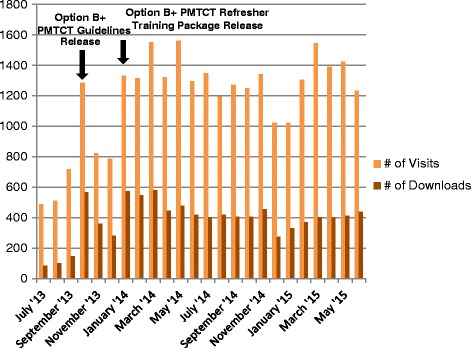
Number of visits to the Tanzania PMTCT webpage. Number of visits to the Prevention of Mother-to-Child Transmission of HIV National Resource Center website and resources downloaded from July 2013 through June 2015

Specific targets were not identified for the expected number of visitors, page views or downloads that should be achieved by the website. It was anticipated that a useful, successfully marketed website would demonstrate increased use over time and that visits would spike when important, new national PMTCT resources were released. After website launch in July 2013, Google Analytics reports showed an increase in visits over the first year that paralleled implementation of promotional activities. Monthly visits spiked by about 70 % during October 2013 and January 2014 in response to the release and promotion of online access to the revised national PMTCT guidelines and training package for Option B+, respectively (Fig. 2). Six-month interval usage statistics, summarized in Table 2, indicate that visits increased by 80 % to a peak of 8384 visits during the second 6 months of year 1, then declined by 9–11 % but remained fairly stable in year 2, for a total of 28,400 website visits over 2 years. Almost one-third (30 %) of visits were from returning visitors, i.e., individuals who had been on the site previously.

**Table 2:** Tanzania PMTCT website statistics. Six-month interval website usage statistics over the first two years of the Tanzania Prevention of Mother-to-Child Transmission of HIV (PMTCT) National Resource Center (NRC)

	July -December 2013	January-June 2014	July-December 2014	January-June 2015
Number of visits	4655	8384	7435	7926
Page views	12,009	20,861	16,793	16,800
Bounce rate %	48 %	50 %	51 %	56 %
Mean visit duration (minutes)	3.95	3.58	2.9	2.8
Returning visitors	29.50 %	32 %	28.90 %	29.30 %
Accessing via mobile device	23.8 %	32.2 %	37.2 %	37.35 %
Number of total and key Tanzania resource downloads				
Total downloads	1543	3049	2367	2863
PMTCT guidelines	356	440	333	286
PMTCT pocket guide	123	483	434	412
PMTCT wall chart	101	39	29	34
PMTCT Refresher Training Manual	--	296	210	138
Guidelines for Management of HIV and AIDS	177	395	369	418

From July 2013 through June 2015, 50 % of website visits originated in Tanzania, with about two-thirds (62 %) from Dar es Salaam, which is mostly an urban area. About one-third (32 %) of Tanzania visits came from unspecified locations, and there were small numbers from Arusha, Mwanza and Zanzibar, which is consistent with limited internet access outside of urban centers. Visits that originated outside of Tanzania reflect regional interest by users in other African countries (16 %), Europe (10 %), the United States (9 %), India and Indonesia (6 %) and others (9 %). Website access using mobile devices increased from about one-quarter of visits during the first six-months to more than one-third of visits in year 2, averaging 34 %.

Overall, there 28,400 visits with 66,463 page views of the website over 2 years. The bounce rate, which indicates the percentage of single page sessions, ranged from 48 to 56 % (see Table 2). Visitors who viewed more than one page, viewed an average of 4 pages/visit during year 1 and 3 pages/visit during year 2. The Swahili version of the website received only 1218 page views. Visit duration averaged 3.15 minutes overall, but did decrease from year 1 to year 2 (see Table 2). However, visit duration may be unreliable as it includes visitors who stopped website activities but didn’t close their browser window. There were a total of 9322 downloads from the site; large documents are available in sections to facilitate access. As shown in Table 2, total downloads doubled in the second half of year 1 following release of Option B+ resources and dropped by 22 % in year 2. Downloads of the national PMTCT guidelines and other PMTCT-specific resources show a similar pattern. Tanzania National Guidelines for the Management of HIV and AIDS downloads now exceed those of PMTCT resources and have remained steady in year 2. Other downloaded resources not listed in the table include smaller sections of the guidelines and training manuals, participant manual for the PMTCT Option B+ Refresher Training Manual, the PMTCT wall chart and dosing chart, national PMTCT indicators, patient record form, etc.

**Fig. 3:**
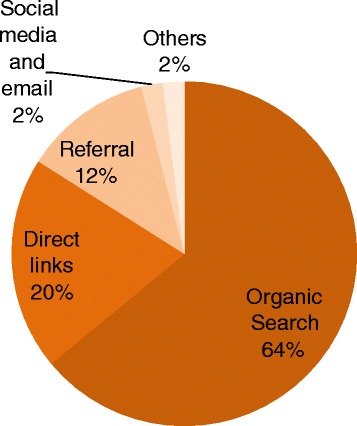
Sources of Tanzania PMTCT webpage traffic. Sources of traffic to the Tanzania Prevention of Mother-to-Child Transmission of HIV National Resource Center website from July 2013 through June 2015.

Figure 3 shows the distribution traffic to the site and provides insights about the effectiveness of promotional strategies. About 20 % of visitors (n = 5845) came directly to the website, which indicate that they typed the website URL directly in their browsers or had it bookmarked. The website URL was included on distributed promotional materials, e.g., post cards, posters, pens and bags. Direct traffic can be used as a proxy indicator about the success of offline website marketing strategies. Twelve percent of traffic came from referrals. This group includes visitors who were referred to the PMTCT NRC website through an inbound link from other websites, e.g., websites of the MOHSW, PMTCT partners, universities and other organizations. Referral traffic shows that crosslinking played a definite role in directing visitors to the PMTCT NRC website. However, the majority (64 %) of visitors (n = 18,066) accessed the website through an organic search, indicating that it was listed on results generated in response to a topic search—commonly through the Google search engine. The PMTCT NRC website is listed first when searching for Tanzania PMTCT on various search engines. This observation demonstrates that the website is well constructed and can be located effectively.

Social media and listserv emails have played a small but important role in promoting use of the PMTCT NRC website, accounting for 2 % (n = 486) of website visitors. The PMTCT website Facebook page has received 238 likes, and website has 94 followers on Twitter, indicators showing that people are interested and are following posted PMTCT updates. As noted previously, following the launch of a social media campaign on Facebook and Twitter and listserv emails in October 2013 to promote the updated national PMTCT guidelines for Option B+, there was 70 % increase in website traffic and the number of resources downloaded increased almost four-fold compared to the previous month (Fig. 2).

## Discussion

The pattern of visits, page views and downloads over the first 2 years of the PMTCT NRC demonstrates successful implementation of the website and its role in dissemination of national PMTCT resources that are needed to support implementation of Option B+. The website focus on PMTCT facilitates site navigation and searches for novice internet users. Large documents are split into sections to facilitate online access and downloads. Importantly, the ability to limit search time and download only needed resources reduces the costs associated with internet use in Tanzania. However, stable or slightly reduced use during year 2 highlights the importance of continued development, promotion and evaluation to foster increased numbers of users, particularly as internet access expands. Future efforts should be directed toward improving the reach and effectiveness of social media, particularly among students and young healthcare workers, in conjunction with listserv emails and other promotional activities.

Discussion with and feedback from the Website Advisory Committee and internal quality reviews were used to refine early versions of the website. Although a link to a static user survey is prominently featured on the home page, responses have been limited. Anecdotal feedback from PMTCT stakeholders and from the website feedback form has been generally positive and helpful. One user commented that “*the website is good—updated with current peer reviewed articles and guidelines”* but suggested adding more information about the current PMTCT situation in Tanzania, including annual data and trends over time. The online interactive discussion forum has had only limited participation, but the Website Advisory Committee feels that it is an important area for future development and has suggested incorporating special updates from the MOHSW, success stories or video clips from partners, etc.

A recent study reported sub-optimal and outdated knowledge about the recommended antiretroviral regimens for PMTCT among healthcare workers in Tanzania [21] and another identified gaps related to related to dissemination of guidelines about HIV and infant feeding [22]. The online PMTCT NRC provides an opportunity to address knowledge deficits by making national PMTCT guidelines and other resources, such as PMTCT training curricula and job aids, more readily available to healthcare workers and health educators. Increasing information access is an important step in improving the implementation and quality of PMTCT services. FXBT Health has begun to include questions about awareness of and access to the website and ability to download resources as part of site visits to PMTCT clinical sites, information that will be useful going forward. Freely available Google Analytics software has been a useful tool in monitoring and evaluating the website. However, other approaches are needed to assess and improve the outcomes and impact of the website, such as focus group discussions, key informant interviews, or surveys of prospective and current users.

The MOHSW has demonstrated commitment to advancing the delivery and effectiveness of PMTCT services and has been instrumental in actively promoting the PMTCT NRC website. Their decision to support the creation of separate website for the PMTCT was based in part on recognition of the need to provide ease of access to online information through a website focused on this topic. A link to the PMTCT NRC is featured on the MOHSW home page, and MOHSW personnel direct individuals to the PMTCT NRC website in response to requests for resources and information about PMTCT. The PMTCT NRC website belongs to the MOHSW but is hosted, managed and updated by FXBT Health, a PMTCT technical assistance partner. It is imperative that key PMTCT stakeholders take ownership of the website in order to increase its credibility and ensure its sustainability over time. PMTCT partners in Tanzania have demonstrated their ownership of and commitment to the website by contributing resources based on their work in different regions and by providing feedback about the website.

The observation that the majority of PMTCT NRC users are concentrated in urban areas is consistent with identified challenges on how to reach those in rural areas with limited internet access [23]. A recent study about internet access and penetration in three African countries, including Tanzania, found that the overall picture with regards to Internet availability is one of rapidly increasing access—particularly into rural and previously under-served areas, falling prices and improving services [24]. These findings suggest that healthcare workers in urban and rural areas will be able to access health information and resources through the internet over time. Our results show an increase in the percentage of PMTCT website users who are using mobile phones to access the PMTCT NRC website. To advance use of e-health resources in resource-limited settings, it is important to develop websites that provide this flexibility in access. Carefully designed, easily navigated websites that address national health issues, services and resources are essential to meeting the needs of novice internet users. The PMTCT NRC website is designed to assure access to information and resources essential to the implementation of revised national PMTCT guidelines and the scale-up of comprehensive PMTCT clinical services in Tanzania. Additional work with stakeholders and constituents is needed to assess current reach of the website among targeted users and identify new strategies to support its use.

## Conclusions

With the successful implementation of the PMTCT NRC website, Tanzania now has centralized, online information designed to address the needs of clinicians, educators, public health professionals and PMTCT program partners in Tanzania. This initial website evaluation shows that an online resource center is feasible in a resource-limited setting like Tanzania despite the challenges of limited Internet availability. However, additional efforts are needed to expand use of the website going forward by improving awareness of and access to the online PMTCT NRC and its resources. Increasing availability and access to the Internet, especially with mobile device technology, brings hope that increasing numbers of healthcare workers and other stakeholders may be able to access important electronic health information resources even in rural areas. A one-stop access point for information through the PMTCT NRC website provides an opportunity for healthcare workers to obtain and download information and resources that are needed to provide quality PMTCT services. Development and promotion of the website should be a continuous and critical process and should use a combination of both online and offline approaches to ensure that the target audience is always prioritized and needs are met. The ongoing involvement and commitment of the MOHSW and key stakeholders, such as the PMTCT partners, are essential to ensure the credibility, growth, effectiveness and sustainability of the PMTCT NRC website. Future evaluations should address the role and impact of the website in improving knowledge about PMTCT and supporting implementation of national PMTCT guidelines and services.
